# Atmospheric CO_2_ Concentration and N Availability Affect the Balance of the Two Photosystems in Mature Leaves of Rice Plants Grown at a Free-Air CO_2_ Enrichment Site

**DOI:** 10.3389/fpls.2020.00786

**Published:** 2020-06-09

**Authors:** Hiroshi Ozaki, Takeshi Tokida, Hirofumi Nakamura, Hidemitsu Sakai, Toshihiro Hasegawa, Ko Noguchi

**Affiliations:** ^1^School of Life Sciences, Tokyo University of Pharmacy and Life Sciences, Hachioji, Japan; ^2^Division of Biogeochemical Cycles, Institute for Agro-Environmental Sciences, Tsukuba, Japan; ^3^Taiyo Keiki Co., Ltd., Toda, Japan; ^4^Division of Climate Change, Institute for Agro-Environmental Sciences, Tsukuba, Japan; ^5^Division of Agro-Environmental Research, Tohoku Agricultural Research Center, Morioka, Japan

**Keywords:** CO_2_ enrichment, FACE, nitrogen, photosystem I, photosystem II, rice (*Oryza sativa*)

## Abstract

Atmospheric CO_2_ concentration ([CO_2_]) has been substantially increasing. Responses of leaf photosynthesis to elevated [CO_2_] have been intensively investigated because leaf photosynthesis is one of the most important determinants of crop yield. The responses of photosynthesis to elevated [CO_2_] can depend on nitrogen (N) availability. Here, we aimed to investigate the significance of the appropriate balance between two photosystems [photosystem I (PSI) and photosystem II (PSII)] under various [CO_2_] and N levels, and thus to clarify if responses of photosynthetic electron transport rates (ETRs) of the two photosystems to elevated [CO_2_] are altered by N availability. Thus, we examined parameters of the two photosystems in mature leaves of rice plants grown under two [CO_2_] levels (ambient and 200 μmol mol^–1^ above ambient) and three N fertilization levels at the Tsukuba free-air CO_2_ enrichment experimental facility in Japan. Responses of ETR of PSII (ETRII) and ETR of PSI (ETRI) to [CO_2_] levels differed among N levels. When moderate levels of N were applied (MN), ETRI was higher under elevated [CO_2_], whereas at high levels of N were applied (HN), both ETRII and ETRI were lower under elevated [CO_2_] compared with ambient [CO_2_]. Under HN, the decreases in ETRII and ETRI under elevated [CO_2_] were due to increases in the non-photochemical quenching of PSII [Y(NPQ)] and the donor side limitation of PSI [Y(ND)], respectively. The relationship between the effective quantum yields of PSI [Y(I)] and PSII [Y(II)] changed under elevated [CO_2_] and low levels of N (LN). Under both conditions, the ratio of Y(I) to Y(II) was higher than under other conditions. The elevated [CO_2_] and low N changed the balance of the two photosystems. This change may be important because it can induce the cyclic electron flow around PSI, leading to induction of non-photochemical quenching to avoid photoinhibition.

## Introduction

Atmospheric CO_2_ concentration ([CO_2_]) has increased substantially since the Industrial Revolution. Since leaf photosynthesis is one of the most important determinants of crop yield, responses of photosynthesis to elevated [CO_2_] have been intensively examined (Long et al., 2004; Ainsworth and Long, 2005; Ainsworth and Rogers, 2007; Leakey et al., 2009a; Xu et al., 2015). For example, long-term elevated [CO_2_] by free-air CO_2_ enrichment (FACE) experiments have been used to stimulate leaf photosynthesis in C_3_ plants (Long et al., 2004; Leakey et al., 2009a). In the case of rice, a meta-analysis revealed that biomass and yield increase by approximately 20% under long-term elevated [CO_2_] (Ainsworth, 2008). However, plants that acclimate to long-term elevated [CO_2_] conditions show lower increases in photosynthesis and yield than expected (Long et al., 2004). In C_3_ species, photosynthesis is usually considered to be limited by the carboxylation capacity of Rubisco (V_cmax_), and/or ribulose-1,5-bisphosphate (RuBP) regeneration rate (J_max_) (Farquhar et al., 1980). In many plant species, including rice, V_cmax_ and J_max_ are decreased by elevated [CO_2_] (Bernacchi et al., 2005; Chen et al., 2005, 2014; Zhang et al., 2008; Hasegawa et al., 2016). Although plants grown under elevated [CO_2_] often show a larger decrease in V_cmax_ compared with J_max_, the limitation of photosynthetic rates under elevated [CO_2_] will shift from V_cmax_ to J_max_. The long-term responses of V_cmax_ and J_max_ to elevated [CO_2_], analyzed in soybean plants at FACE site, showed a shift of the limitation from V_cmax_ to J_max_ (Bernacchi et al., 2005). In a durum wheat cultivar that has a low harvest index, both V_cmax_ and J_max_ decreased, but the degree of decreases in V_cmax_ was larger than that in J_max_, leading to an increase in the ratio of J_max_ to V_cmax_ under elevated [CO_2_] (Aranjuelo et al., 2013). The decreases in V_cmax_ and J_max_ occurred not only at FACE sites but also at a natural CO_2_ spring where [CO_2_] is consistently high (Saban et al., 2019). Plants grown at the CO_2_ spring exhibited a larger decrease in V_cmax_ compared to J_max_ (Saban et al., 2019).

J_max_ is related to the whole photosynthetic electron transport system. Several studies examined the effective quantum yield of photosystem II (PSII) [Y(II)] and/or electron transport rate of PSII (ETRII) in leaves grown under long-term elevated [CO_2_] using chlorophyll (Chl) fluorescence measurements. Habash et al. (1995) reported that wheat plants grown under elevated [CO_2_] showed higher Y(II) than those grown under ambient [CO_2_]. Cousins et al. (2001) examined responses of Y(II) to long-term [CO_2_] in *Sorghum bicolor* grown at FACE sites. A small decrease in Y(II) was observed under low measurement CO_2_ condition in *Sorghum* plants grown at FACE sites. In contrasts, responses of photosystem I (PSI) to long-term elevated [CO_2_] have rarely been examined. Pan et al. (2018) examined effects of elevated [CO_2_] and heat stress to Y(II) and the effective quantum yields of PSI [Y(I)] in tomato plants. They showed that long-term elevated [CO_2_] ameliorated the decrease in both Y(II) and Y(I) by heat stress.

The photosynthetic electron transport through both PSII and PSI supplies ATP and NADPH. This supply should be matched to their demands in downstream metabolism such as the Calvin cycle and the photorespiratory pathway (Noctor and Foyer, 2000). The cyclic electron transport around PSI (CEF-PSI) in terrestrial plants consists of two partially redundant pathways, the ferredoxin (Fd)-dependent CEF-PSI and the NAD(P)H dehydrogenase (NDH)-dependent pathways, and can balance the ratio of ATP to NADPH production (Shikanai, 2014). Therefore, the response of CEF-PSI to elevated [CO_2_] is important for balancing supply and demand. Also, the balance between two photosystems, PSII and PSI, is important to suppress the generation of reactive oxygen species (ROS) in the photosynthetic electron transport. However, few studies have examined the balance between the two photosystems in leaves grown under elevated [CO_2_].

Nitrogen (N) availability intensely affects responses of leaf photosynthesis to elevated [CO_2_] (Xu et al., 2015). Significant decreases in V_cmax_ and J_max_ under elevated [CO_2_] were observed in wheat leaves under low N fertilization (Miglietta et al., 1996). In leaves of *Arabidopsis thaliana* under elevated [CO_2_], the carbohydrate accumulation and down regulation of photosynthetic genes were observed (Cheng et al., 1998). When plants with low sink capacity are grown under low N and elevated [CO_2_], carbohydrates accumulate in leaves, and the sugar repression leads to the down regulation of photosynthetic gene expression (Drake et al., 1997; Rogers et al., 1998; Long et al., 2004). The decrease in photosynthesis under low N and elevated [CO_2_] is also attributed to decreased concentrations of leaf N (Nakano et al., 1997; Seneweera et al., 2011). Since plants invest a large quantity of N into photosynthetic enzymes, the decrease in leaf N under elevated [CO_2_] can directly lead to decreased photosynthetic rates. However, while many studies have examined the responses of ribulose 1,5-bisphosphatecarboxylase/oxygenase (Rubisco) and V_cmax_ to low N and elevated [CO_2_], few studies have examined the response of photosynthetic electron transport to low N and elevated [CO_2_]. Therefore, it is important to clarify the responses of photosynthetic electron flow through the two photosystems to elevated [CO_2_], and how these responses differ depending on N availability. This is because the appropriate balance between the two photosystems and the response of CEF-PSI are important not only for relevant supply of NADPH and ATP but also for suppression of ROS production under various environmental conditions.

In this study, we aimed to investigate the significance of the appropriate balance between two photosystems under various [CO_2_] and N levels, and thus addressed two following hypotheses. (i) The linear electron transport rate (ETR) responds to elevated [CO_2_], and its response depends on N availability. (ii) The response of PSI to elevated [CO_2_] and N availability is different from the response of PSII, and the balance between the two photosystems differs depending on [CO_2_] or N availability. To address the above hypotheses, we examined the two photosystems in mature leaves of a *japonica* rice variety (Koshihikari) grown under two [CO_2_] levels (ambient and 200 μmol mol^–1^ above ambient) and three N fertilization levels at the Tsukuba FACE experimental site in central Japan. At this FACE site, growth, photosynthesis and yield have been examined for many rice varieties including Koshihikari (Hasegawa et al., 2013; Chen et al., 2014; Usui et al., 2014; Ikawa et al., 2018; Sakai et al., 2019). Koshihikari is one of the standard *japonica* varieties and the most widely planted variety in Japan. We measured the photochemical parameters of the two photosystems in Koshihikari leaves at a vegetative growth stage, and determined the N and carbon (C) contents of the leaves. The results indicated that the balance between the two photosystems changed under elevated [CO_2_] and low levels of N (LN). Under both conditions, the ratio of Y(I) to Y(II) was higher than under other conditions.

## Materials and Methods

### Site Description

The study was conducted at the Tsukuba FACE experimental facility in Tsukubamirai, Ibaraki, Japan (35°58′ N, 139°60′ E) in 2017. There were four pairs of ambient and elevated [CO_2_] plots at the site. We used two of the four pairs of plots. The average ambient [CO_2_] at the site across the entire growing season (June–September) and day-to-day SD was 391 ± 12.8 μmol mol^–1^. The CO_2_ enrichment was performed only in the daytime. The target concentration of the elevated [CO_2_] treatment was 200 μmol mol^–1^ above ambient [CO_2_], and the actual season-long mean [CO_2_] and day-to-day SD in the FACE plots was 585 ± 16.3 μmol mol^–1^. Previously published articles have provided further details of the experimental site set-up and CO_2_ control performance (Nakamura et al., 2012) and soil chemical properties (Hasegawa et al., 2013).

### Plant Materials

We used a *japonica* rice (*Oryza sativa* L.; variety, Koshihikari). Three-week-old seedlings were transplanted into the experimental plots on May 24 and 25 in 2017. Plants received fertilizers prior to planting at a rate of 4.36 g m^–2^ of P and 8.3 g m^–2^ of K, as described in Hasegawa et al. (2013). Under the moderate (standard) N treatment (MN), 8 g m^–2^ of N was applied. For the low and high N treatments (LN and HN), 0 and 12 g m^–2^ were applied, respectively. Further details of N fertilization can be found in Hasegawa et al. (2019). We sampled the uppermost fully expanded leaves at the vegetative stage (from June 19 to July 6). Four biological replications were used for each experiment.

### Measurement of Chlorophyll Fluorescence and P700 Absorption Using a Dual-PAM-100 Measuring System

Leaves were sampled in each plot, and measured in the cabin at the site. Measurements were taken on detached leaves at a room temperature and ambient CO_2_ concentration. Chl fluorescence and absorption changes at 830 nm were simultaneously measured using a Dual-PAM-100 (Walz, Effeltrich, Germany). The Chl fluorescence parameters were calculated according to the methods developed by Genty et al. (1989); Baker et al. (2007), and Kono et al. (2014). The Y(II) was calculated as (F_m_′−F_s_)/F_m_′, where F_m_′ and F_s_ are the maximum fluorescence level and the steady-state fluorescence level under actinic light (AL), respectively. Two other PSII quantum yields, Y(NPQ) and Y(NO), which represent the regulated and non-regulated energy dissipation at PSII centers, were calculated as F_s_/F_m_′−F_s_/F_m_ and F_s_/F_m_, respectively. F_m_ is the maximum fluorescence level in the dark-adapted state. The fraction of PSII centers that are in the open state, qL, was calculated as (F_m_′−F_s_)(F_o_′/ F_s_)/(F_m_′−F_o_′). Additionally, F_o_′, the minimal fluorescence yield under AL, was estimated as described by Oxborough and Baker (1997) [i.e., F_o_/(F_v_/F_m_ + F_o_/F_m_′)]. The ETR through PSII was calculated as ETRII = Y(II) × PPFD × 0.84 × 0.5, where 0.84 and 0.5 were based on the assumption that leaves absorb 84% of incident photons and that 50% of these photons are absorbed by PSII.

In the Dual-PAM-100, P700^+^ was monitored as the difference between the absorptions at 830 and 875 nm in the transmission mode. We estimated PSI parameters, including photochemical quantum yield [Y(I)], non-photochemical quantum yield due to donor side limitations [Y(ND)], non-photochemical quantum yield due to acceptor side limitations [Y(NA)], and the PSI electron transport rate (ETRI), as described by Baker et al. (2007) and Kono et al. (2014). We estimated ETRI based on the assumption that leaves absorb 84% of incident photons and that 50% of these photons are absorbed by PSI. The steady-state rate of CEF-PSI was estimated by subtracting ETRII from ETRI, when each rate was simultaneously estimated (Dahal et al., 2014; Yamada et al., 2020).

The minimum fluorescence level in the dark-adapted state (F_o_), F_m_, and the maximum P700 signal (fully oxidized P700) in darkness (P_m_) were measured after a 30-min incubation in darkness. Subsequently, we illuminated leaves at the strong AL (1599 μmol photons m^–2^ s^–1^) to activate the photosynthesis for 28 min, and then measured Chl fluorescence and P700 absorption at 978, 602, 356, and 131 μmol photons m^–2^ s^–1^ of AL to generate photosynthetic light-response curves.

### Determinations of Leaf Dry Weight, and Contents of Carbon and Nitrogen

After the Chl fluorescence and P700 absorption change were measured, leaf segments were dried at 80°C for more than 48 h and then weighed to measure leaf mass per area (LMA). After the LMA was determined, C and N contents of the samples were measured with an MT-700 Mark 2 CN analyzer (Yanaco, Kyoto, Japan).

### Statistical Analyses

An analysis of variance, Tukey–Kramer multiple comparison test, analysis of covariance (ANCOVA) and correlation analysis were conducted using the statistical software R (R Core Team, 2016). The analyzed data in Figures 1–5 and Supplementary Figures S1–S6 are summarized in Supplementary Table S1. Statistical significance was noted if *p* < 0.1.

**FIGURE 1:**
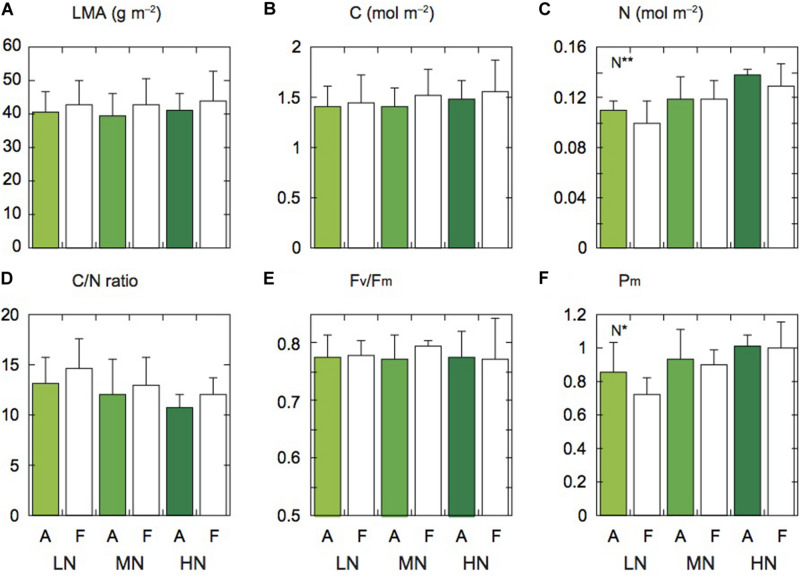
Leaf characteristics of rice plants. Leaf mass per area (LMA; **A**), carbon content (C; **B**), nitrogen content (N; **C**), ratio of carbon to nitrogen contents (C/N; **D**), F_v_/F_m_ **(E)**, and P_m_ **(F)** under low (LN), moderate (MN) and high nitrogen levels (HN). Panels **(A,F)** denote the data of leaves under ambient and elevated [CO_2_] levels. Data are presented as the mean ± standard deviation (*n* = 4). Statistical results are also shown (*p < 0.05, **p < 0.01).

**FIGURE 2:**
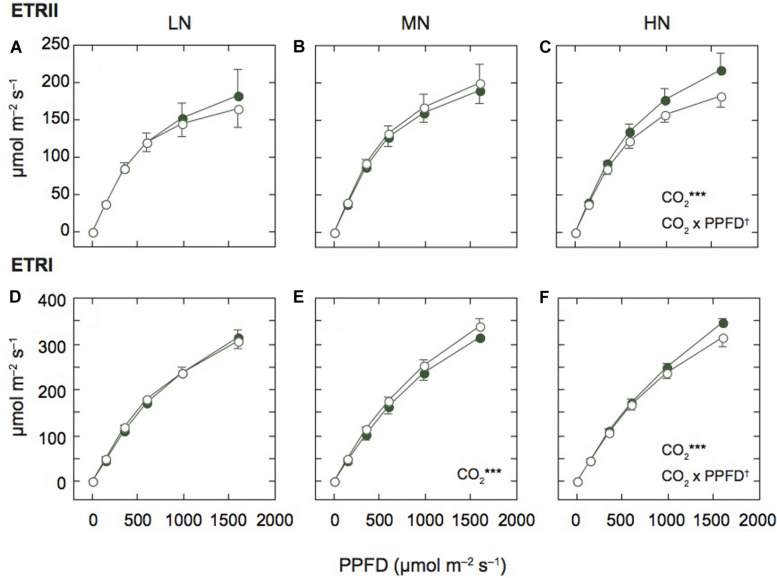
Light-response curves of photosynthetic electron transport in mature leaves of rice plants. The electron transport rate in PSII (ETRII; **A–C**) and PSI (ETRI; **D–F**) under low (LN; **A,D**), moderate (MN; **B,E**) and high nitrogen levels (HN; **C,F**). Closed and open symbols denote the data of leaves under ambient and elevated [CO_2_] levels. Data are presented as the mean ± standard deviation (*n* = 4). Statistical results are also shown (^†^*p* < 0.1, *p < 0.05, **p < 0.01, ***p < 0.001). CO_2_ and CO_2_ × PPFD denote the effects of CO_2_ concentration and interaction between CO_2_ and PPFD, respectively. Statistical results of effects of PPFD were less than 0.001 under all N levels.

**FIGURE 3:**
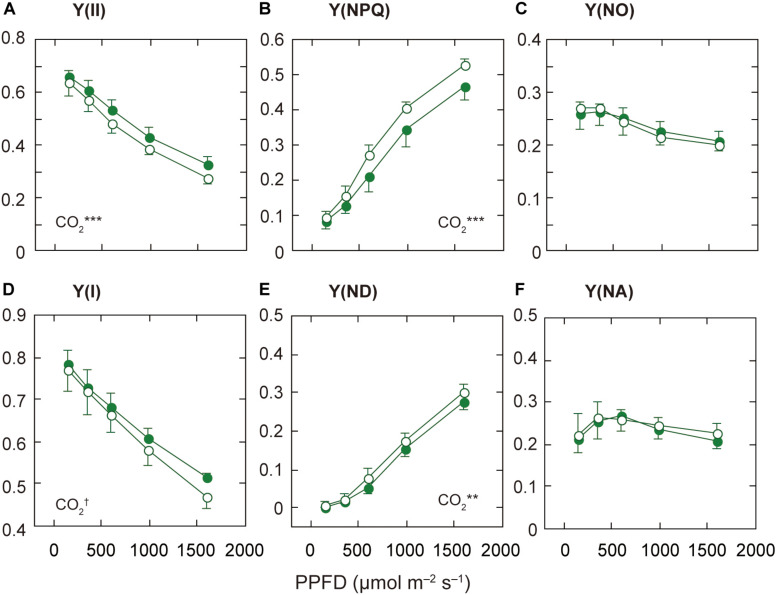
Light-response curves of PSII and PSI parameters in mature leaves of rice plants grown under a high nitrogen level (HN). The effective quantum yield of PSII [Y(II); **A**], the quantum yield of regulated [Y(NPQ); **B**] and non-regulated [Y(NO); **C**] energy dissipation in PSII. The effective quantum yield of PSI [Y(I); **D**], the non-photochemical quantum yield due to donor side limitations [Y(ND); **E**], and the non-photochemical quantum yield due to acceptor side limitations [Y(NA); **F**]. Statistical results of effects of PPFD are less than 0.05. For other details, see the legend of Figure 2.

**FIGURE 4:**
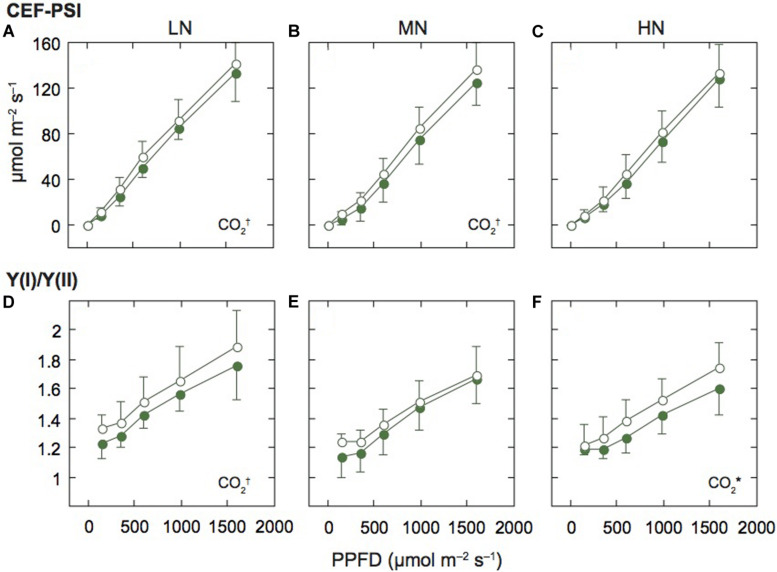
Light-response curves of the rate of cyclic electron flow around PSI and the ratio of PSI to PSII activities in mature leaves of rice plants. The rate of the cyclic electron flow around PSI (CEF-PSI; **A–C**), and the ratio of effective quantum yield of PSI [Y(I)] to effective quantum yield of PSII [Y(II)] [Y(I)/Y(II); **D–F**] under low (LN; **A,D**), moderate (MN; **B,E**) and high nitrogen levels (HN; **C,F**). For other details, see the legend of Figure 2.

**FIGURE 5:**
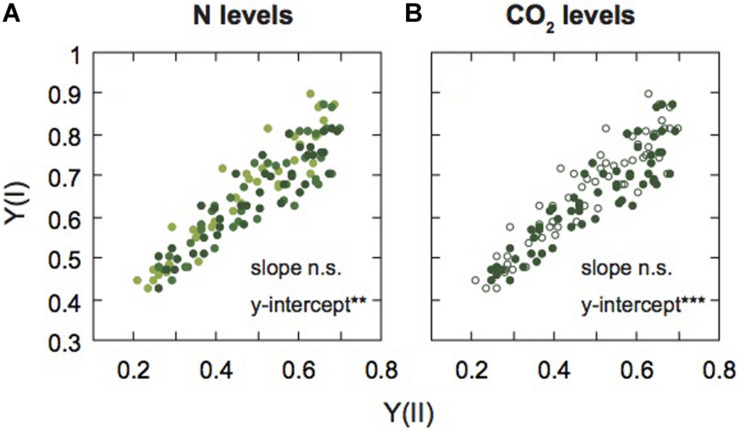
Relationship between PSI and PSII activities in mature leaves of rice plants. The relationship between effective quantum yield of PSI [Y(I)] and effective quantum yield of PSII [Y(II)] are shown under each N level **(A)** and each [CO_2_] level **(B)**. Light green, medium green, and dark green symbols denote low (LN), moderate (MN), and high nitrogen levels (HN), respectively **(A)**. Closed and open symbols denote the data of leaves under ambient and elevated [CO_2_] levels **(B)**. Statistical significance in slopes and y-intercepts using ANCOVA are also shown (**p < 0.01, ***p < 0.001).

## Results

### Leaf Characteristics

There were no significant differences in LMA and leaf C content between the two [CO_2_] levels and among the three N levels, but these parameters under elevated [CO_2_] tended to be lower compared with those under ambient [CO_2_] (Figures 1A,B). Leaf N content significantly differed among the three N levels (Figure 1C). The N content under elevated [CO_2_] was lower than under ambient [CO_2_], but the difference was insignificant. In our previous study at the same FACE site, the ratio of C to N (C/N ratio) of Koshihikari leaves was higher under elevated [CO_2_] compared with under ambient [CO_2_] (Noguchi et al., 2018). In this study, this ratio tended to be high under elevated [CO_2_] (Figure 1D). This ratio was also different among the N levels, but the difference was insignificant.

Since low N often induces PSII photoinhibition in leaves (Noguchi and Terashima, 2006; Kumagai et al., 2009), we measured the maximum photochemical quantum yield of PSII (F_v_/F_m_). The F_v_/F_m_ values were not different between the two [CO_2_] levels and among the three N levels (Figure 1E). In Koshihikari leaves, PSII photoinhibition was not observed under low N (LN). We also measured the maximum fully oxidized P700 in darkness (P_m_). The P_m_ value of LN leaves was significantly lower than that of the other N levels (Figure 1F). The PSI contents may be lower in LN leaves. The P_m_ values were not different between the two [CO_2_] levels.

### Photosynthetic Electron Transport Rates Through PSII and PSI Were Decreased by Elevated [CO_2_] Under the HN Condition

Since the N contents and P_m_ values were different among the N levels, we compared the light response curves of ETRII and ETRI between the two [CO_2_] levels within each N level (Figure 2). Under MN, ETRII were not different between the two [CO_2_] levels, but ETRI of leaves under elevated [CO_2_] was significantly higher than that under ambient [CO_2_]. Under LN, ETRII of leaves under elevated [CO_2_] tended to be lower than that under ambient [CO_2_], but the difference was insignificant. ETRI of leaves under LN were similar between the two [CO_2_] levels. In contrast, under HN, both ETRII and ETRI were significantly lower in leaves under elevated [CO_2_] compared with those under ambient [CO_2_]. Previous studies reported that photosynthetic rates were decreased by elevated [CO_2_] in leaves under low N fertilization (e.g., Miglietta et al., 1996). However, in this study, both ETRs were decreased by elevated [CO_2_] only under HN.

We also compared ETRs at the highest AL intensity among the N levels (Supplementary Figures S1A,B). ETRI was different among the N levels (*p* < 0.1), and the interaction between N and [CO_2_] levels was significant.

### Under the High N Condition, the Decrease in Y(II) Is Related to the Increase in Y(NPQ) Under Elevated [CO_2_]

Since the responses of ETRII to elevated [CO_2_] were different among the N levels, we examined the light-dependence of the PSII parameters, Y(II), Y(NPQ), and Y(NO), based on the Chl fluorescence data. The decrease in Y(II) is due to increases in Y(NPQ) and/or Y(NO) (Baker et al., 2007). Under LN and MN, Y(II) did not differ between the [CO_2_] levels, but under HN, Y(II) was lower under elevated [CO_2_] (Figure 3A and Supplementary Figures S2A,B). This decrease in Y(II) was related to the increase in Y(NPQ), the regulated energy dissipation at PSII centers, under elevated [CO_2_] (Figure 3B). Under the other N conditions, Y(NPQ) was not significantly different between the two [CO_2_] levels (Supplementary Figures S2C,D). On the other hand, the difference in Y(NO) between the two [CO_2_] levels was small under all N levels, although Y(NO) was significantly different between the two [CO_2_] levels only under MN (Figure 3C and Supplementary Figures S2E,F). The relationship between Y(II) and Y(NO) was not well correlated (Supplementary Figures S3D–F). In contrast, the relationship between Y(II) and Y(NPQ) was well correlated irrespective of N level (Supplementary Figures S3A–C). This good correlation suggests that the dependence of Y(II) on measurement light intensity is mainly determined by that of Y(NPQ).

The qL, the fraction of PSII centers that are in the open state, was not different between the two [CO_2_] levels under any N level (data not shown). The decrease in ETRII under elevated [CO_2_] and HN may not be determined by the limited electron flow downstream of PSII. The decrease in ETRII under elevated [CO_2_] and HN is mainly determined by the increase in non-photochemical quenching.

We compared the PSII parameters among N levels (Supplementary Figures S1D–F). Under the ambient [CO_2_], Y(II) under LN was lower than that under HN, but the difference was insignificant. The other parameters, Y(NPQ) and Y(NO), were similar among the N levels.

### Under the High N Condition, the Decrease in Y(I) Is Related to the Increase in Donor-Side Limitation Under Elevated [CO_2_]

Next, we examined the light-dependence of the PSI parameters based on the P700 absorption data. The decrease in Y(I) is caused by increases in non-photochemical quantum yield due to donor side limitations [Y(ND)] and/or non-photochemical quantum yield due to acceptor side limitations [Y(NA)] (Baker et al., 2007). Under MN and HN, Y(I) was significantly different between the two [CO_2_] levels, although the responses to elevated [CO_2_] were different between the two N levels (Figure 3D and Supplementary Figure S4B). Under MN, the difference in Y(I) between the two [CO_2_] levels was mainly dependent on that in Y(NA) (Supplementary Figure S4D). Under MN, the decrease in Y(NA) under elevated [CO_2_] determined the increase in Y(I). Under MN, elevated [CO_2_] may increase the components downstream of PSI, leading to a decrease in Y(NA). Under LN, the difference in Y(NA) was small but significant (Supplementary Figure S4E). The decrease in Y(NA) under elevated [CO_2_] determined the increase in Y(I) under LN. In contrasts, under HN, Y(NA) was not different between the two [CO_2_] levels (Figure 3F). Under HN, the acceptor side limitations of PSI may be similar between the two [CO_2_] levels, but elevated [CO_2_] may lead to H^+^ accumulation in the thylakoid lumen. This H^+^ accumulation may affect the increase in Y(ND) in leaves under elevated [CO_2_] and HN (Figure 3E). We examined the relationships between Y(I) and Y(ND) and between Y(I) and Y(NA) (Supplementary Figure S5). Y(I) and Y(ND) were well correlated irrespective of N level (Supplementary Figures S5A–C). This good correlation suggests that the dependence of Y(I) on measurement light intensity is mainly determined by that of Y(ND).

We investigated the PSI parameters at the highest AL intensity among N levels (Supplementary Figures S1G–I). Y(I) differed among the N levels (*p* < 0.1). The Y(ND) significantly differed among the N levels, and was highest under LN (Supplementary Figure S1H). The low N availability induced the donor-side limitation of PSI.

### The Relationship Between PSII and PSI Changed Under LN and Elevated [CO_2_]

We assumed that ETRII equals the linear ETR, and estimated the rate of CEF-PSI by subtracting ETRII from ETRI. Under all N levels, CEF-PSI increased with the increase in measurement light intensity (Figures 4A–C). Under HN, CEF-PSI was similar between the two [CO_2_] levels. On the other hand, under MN and LN, CEF-PSI under elevated [CO_2_] was higher than that under ambient [CO_2_] (*p* < 0.1). We also examined the ratio of Y(I) to Y(II) [Y(I)/Y(II)], similar parameter to CEF-PSI. The Y(I)/Y(II) value increased with the increase in measurement light intensity (Figures 4D–F). Under LN and HN, Y(I)/Y(II) under elevated [CO_2_] was higher than that under ambient [CO_2_] (*p* = 0.0715 for LN and *p* = 0.0482 for HN). Both parameters suggested that the activity of PSI is higher than that of PSII under elevated [CO_2_].

Next, to compare the balance between PSI and PSII under the two [CO_2_] levels and the three N levels, we examined the relationship between Y(I) and Y(II) (Figure 5). The data were divided into different N levels (Figure 5A) and different [CO_2_] levels (Figure 5B). We analyzed the statistical significance between slopes and y-intercepts using ANCOVA. There were no significant differences in slopes between the [CO_2_] levels and among the N levels. However, significant differences in the y-intercepts between the [CO_2_] levels and among the N levels were observed (Supplementary Table S1). The y-intercept of the regression line under LN and elevated [CO_2_] were higher than the other conditions, that is Y(I) was higher than Y(II) under LN and elevated [CO_2_]. Under low N and elevated [CO_2_], ETRII is suppressed compared with ETRI, leading to increases in Y(I)/Y(II). The Y(NPQ) was increased, but not Y(NO) under conditions where ETRII was suppressed (Figure 3), suggesting that excess energy may be safely dissipated by non-photochemical quenching. Under LN and elevated [CO_2_], CEF-PSI was up-regulated, leading to the suppression of the increase in Y(NA). An increase in Y(NA) can induce the production of ROS in PSI (Sejima et al., 2014). The changes in balance between the two photosystems may suppress ROS production under LN and elevated [CO_2_].

## Discussion

In this study, we aimed to clarify the responses of ETRs to elevated [CO_2_] and if these responses change depending on N availability. We found that the responses of linear ETR of Koshihikari leaves to elevated [CO_2_] changed among different N levels. Under HN, the linear ETR was lower under elevated [CO_2_] than ambient [CO_2_]. Under HN, ETRI was also lower under elevated [CO_2_]. The decreases in both ETRs were due to the increases in non-photochemical quenching in PSII and the donor side limitation of PSI, respectively. We also found that the degree of the decrease under elevated [CO_2_] was different for both ETRII and ETRI, and thereby the relationship between Y(I) and Y(II) changed under elevated [CO_2_]. The increases in the Y(I)/Y(II) ratio imply the induction of CEF-PSI, leading to H^+^ accumulation in the lumen of the thylakoid membrane and induction of non-photochemical quenching (Miyake, 2010).

### The Response of Photosynthetic Linear Electron Transport Rate to Elevated [CO_2_] Differed Depending on N Availability

In this study, we assumed that the linear ETR equals ETRII. The responses of ETRII to elevated [CO_2_] were different depending on N level (Figure 2). The photosynthetic electron transport affects the J_max_ value that is estimated from the A-Ci curve. In soybean leaves in a FACE experiment, J_max_ did not show any difference between two [CO_2_] levels (Bernacchi et al., 2005). Under MN in the same FACE site as this study, J_max_ was significantly decreased in rice leaves at the booting and grain-filling stages under elevated [CO_2_] (Chen et al., 2014). The previous experiments including the same FACE site showed that J_max_ is strongly related to leaf N content in rice leaves (Hasegawa et al., 2016). In this study, both ETRs were significantly correlated with leaf N (Supplementary Figure S6). Even under HN, the decrease in leaf N may affect the decrease in ETR under elevated [CO_2_]. In an experiments where seven C_3_ grassland species were exposed to elevated [CO_2_] at a FACE site, three forb species exhibited a decrease in both V_cmax_ and J_max_ in response to elevated [CO_2_], especially under high N condition, whereas four C_3_ grasses did not (Crous et al., 2010). In their study, the grasses had higher root biomass and allocated more biomass to roots under elevated [CO_2_] but the forbs did not. The rice variety used in our study, Koshihikari, was shown to have a larger decrease in J_max_ than an *indica* variety, Takanari (Chen et al., 2014). Takanari had more below-ground biomass than Koshihikari, and had higher yield in response to elevated [CO_2_] (Hasegawa et al., 2013). Even under HN, elevated [CO_2_] may induce the sink limitation, leading to the down regulation of photosynthesis and decreases in leaf N in some plant species including the Koshihikari variety.

Previous studies have reported that the transcripts of genes related to photosynthetic electron transport components are down-regulated under elevated [CO_2_]. Leakey et al. (2009b) showed the down-regulation of these genes in leaves of soybean plants grown under elevated [CO_2_]. Genes related to the RuBP regeneration process were also down-regulated in leaves of *A. thaliana* and rice plants under elevated [CO_2_] compared with under ambient [CO_2_] (Li et al., 2008; Fukayama et al., 2011). In rice leaves under elevated [CO_2_] in a FACE experiment, the amount of cytochrome f protein, the photosynthetic ETR, and J_max_ were lower compared with in leaves under ambient [CO_2_] (Zhang et al., 2008). In this study, we did not examine whether the changes in ETR were related to those in transcripts related to photosynthetic electron transport components, but the degree of down regulation of these transcripts could differ depending on N availability. The responses of P_m_ value to elevated [CO_2_] were different among the N levels (Figure 1F), suggesting that the degree of changes in PSI content may be different among the N levels.

### Responses of the Balance Between PSII and PSI to Elevated [CO_2_]

The response of Y(I) to elevated [CO_2_] differed depending on N level (Figure 3 and Supplementary Figure S4). Under HN, elevated [CO_2_] decreased Y(I) due to the increase in Y(ND). In some cases, a decrease in Y(I) is related to a decrease in P700 content, which can be estimated from P_m_ (Sejima et al., 2014). In this study, because Y(I) was not correlated with P_m_ (*r* = 0.239, *p* = 0.261), the decrease in Y(I) may be determined by the increase in Y(ND), but not the decrease in P_m_. Under MN, elevated [CO_2_] increased Y(I) due to the decrease in Y(NA). This suggests the decrease in the limitation downstream of PSI under this condition. An increase in Y(NA) can induce the generation of hydroxyl radical, one type of ROS, in PSI (Sejima et al., 2014; Shimakawa and Miyake, 2018). In this study, the ROS production in PSI may be lower because Y(NA) was low under all condition. We cannot explain how Y(NA) value was lower under MN and elevated [CO_2_], but the increase in CEF-PSI under elevated [CO_2_] may be related to the decrease in Y(NA). The mutant of Fd-dependent CEF-PSI, *pgr5*, had higher Y(NA) than a wild-type even under the low measurement light intensity (Kono et al., 2014).

Under HN, Y(II) was lower under elevated [CO_2_] due to the increased in Y(NPQ). Y(NO) did not change under elevated [CO_2_] regardless of N level, and it was consistently low (Figure 3). When Y(NO) is increased, charge recombination reactions in PSII are expected to lead to the triplet state of Chl (Telfer, 2014). The triplet Chl can react with O_2_ to produce harmful singlet oxygen in PSII (Müller et al., 2001). In the present study, the decrease in Y(II) was coupled with an increase in Y(NPQ), which effectively resulted in low Y(NO). Under HN, the donor-side limitation in PSI, Y(ND), was higher under elevated [CO_2_]. The increase in the limitation may be induced by H^+^ accumulation in the thylakoid lumen. The acidification of the lumen suppresses the electron flow through the cytochrome b_6_f complex (Tikhonov, 2014), and induces energy-dependent non-photochemical quenching (Baker et al., 2007). In this study, the underlying mechanisms in H^+^ accumulation under HN and elevated [CO_2_] are still unknown. Under these conditions, the decreases in consumption of ATP and NADPH due to the decreased flux in the Calvin cycle may limit photosynthetic electron transport.

Elevated [CO_2_] induces an increase in carboxylase activity and suppression of the oxygenase activity of Rubisco, leading to increases in CO_2_ fixation rates compared with photorespiratory rates (Long et al., 2004). The photorespiratory pathway requires a higher ratio of ATP to NADPH compared with the Calvin cycle (Kramer and Evans, 2011). Since CEF-PSI can supply ATP relative to NADPH, the requirement of CEF-PSI may be lower under elevated [CO_2_] than ambient [CO_2_]. In a study with tobacco plants grown in a growth cabinet, the rate of CEF-PSI was lower under 1000 μmol mol^–1^ [CO_2_] than under 400 μmol mol^–1^ [CO_2_] (Dahal and Vanlerberghe, 2018). In contrast, in this study, the Y(I)/Y(II) and CEF-PSI were higher under elevated [CO_2_] than ambient [CO_2_]. We cannot explain the different responses of CEF-PSI to elevated [CO_2_] between the two studies, but in our study, plants grew under lower [CO_2_] and more fluctuating light environments than the plants in their experiments in a growth cabinet. Under the fluctuating light environments, CEF-PSI may be important to avoid overreduction of photosynthetic electron transport (Kono et al., 2014; Yamori and Shikanai, 2016; Yamori et al., 2016). In the FACE site, the induction of CEF-PSI may be more important because the H^+^ accumulation in the lumen by high CEF-PSI can induce the photosynthetic control and the induction of non-photochemical quenching, leading to avoidance of photoinhibition.

### Effect of N Availability on the Photosystems

The ETRI was lower under low N. The decrease in Y(I) was accompanied by an increase in Y(ND). Also, ETRII tented to be lower under low N and ambient [CO_2_] (Supplementary Figure S1). A similar decrease in ETRII has been shown in many plant species including rice (de Groot et al., 2003; Ushio et al., 2003; Miyake et al., 2005). In tomato leaves, both Y(I) and Y(II) were lower under low N, and the ratio between the two parameters was not affected by N level (de Groot et al., 2003). A similar result was shown in the leaves of tobacco that were grown under two levels of N fertilizations (Miyake et al., 2005). Our results showed the balance between the two photosystems was altered by N availability (Figure 5A). We cannot explain this discrepancy, but in the previous studies mentioned, plants were cultivated in growth cabinets. The fluctuating light environment in the FACE system may affect the balance between the two photosystems under different N availabilities.

## Data Availability Statement

The datasets generated for this study are available on request to the corresponding author.

## Author Contributions

HO, HS, and KN designed the research. HO and KN performed the experiments and analyzed the data. TT, HN, HS, and TH controlled and regulated paddy field conditions and plant qualities. HO, TT, HN, HS, TH, and KN wrote the manuscript.

## Conflict of Interest

HN was employed by the company Taiyo Keiki Co., Ltd. The remaining authors declare that the research was conducted in the absence of any commercial or financial relationships that could be construed as a potential conflict of interest.
