# Supplementary material for: Atmospheric CO2 Concentration and N Availability Affect the Balance of the Two Photosystems in Mature Leaves of Rice Plants Grown at a Free-Air CO2 Enrichment Site
Source: Front Plant Sci. 2020 Jun 9;11:786. doi: 10.3389/fpls.2020.00786 (PMC7296123; doi:10.3389/fpls.2020.00786)
Supplement: Supplementary file 1 [file Data_Sheet_1.docx]

**Atmospheric CO_2_ concentration and N availability affect the balance of two photosystems in mature leaves of rice plants grown at a free-air CO_2_ enrichment site**

**Supplementary Figure S1**

Parameters of photosynthetic electron transport in rice leaves. The electron transport rate in PSII (ETRII, A), PSI (ETRI, B) and the cyclic electron flow around PSI (CEF-PSI, C). The effective quantum yield of PSII [Y(II), D], the quantum yield of regulated [Y(NPQ), E] and non-regulated [Y(NO), F] energy dissipation in PSII. The effective quantum yield of PSI [Y(I), G], the non-photochemical quantum yield due to donor side limitations [Y(ND), H], and the non-photochemical quantum yield due to acceptor side limitations [Y(NA), I]. LN, MN and HN denote the data of leaves under low (LN), moderate (MN) and high nitrogen levels (HN). A and F denote the data of leaves under ambient and elevated [CO_2_] levels. All data were measured at 1599 μmol photons m^−2^ s^−1^. Data are presented as the mean ± standard deviation (n = 4). Statistical results are also shown (†*p* < 0.1, **p* < 0.05, ***p* < 0.01).

**Supplementary Figure S2**

Light-response curves of PSII parameters in mature leaves of rice plants. The effective quantum yield of PSII [Y(II), A, B], the quantum yield of regulated [Y(NPQ), C, D] and non-regulated [Y(NO), E, F] energy dissipation in PSII under low (LN, A, C, E) and middle nitrogen levels (MN, B, D, F). Closed and open symbols denote the data of leaves under ambient and elevated [CO_2_] levels. Data are presented as the mean ± standard deviation (n = 4). Statistical results are also shown (†*p* < 0.1, * *p* < 0.05, ** *p* < 0.01, *** *p* < 0.001). CO_2_ and CO_2_ x PPFD denote the effects of CO_2_ concentration and interaction between CO_2_ and PPFD, respectively. Statistical results of effects of PPFD were less than 0.001 at all N levels.

**Supplementary Figure S3**

Relationships among parameters of PSII. The relationship between the effective quantum yield of PSII [Y(II)] and the quantum yield of regulated energy dissipation in PSII [Y(NPQ)] (A-C), and between Y(II) and non-regulated energy dissipation in PSII [Y(NO)] (D-F) under low (LN, A, D), moderate (MN, B, E) and high nitrogen levels (HN C, F). For other details, see the legend of Fig. 2.

**Supplementary Figure S4**

Light-response curves of PSI parameters in mature leaves of rice plants. The effective quantum yield of PSI [Y(I), A, B], the non-photochemical quantum yield due to donor side limitations [Y(ND), C, D], and the non-photochemical quantum yield due to acceptor side limitations [Y(NA), E, F] under low (LN, A, C, G) and moderate nitrogen levels (MN, B, D, F). Statistical results of effects of PPFD were less than 0.001 in Y(I) and Y(ND) under all N levels. For other details, see the legend of Supplementary Fig. 2.

**Supplementary Figure S5**

Relationships among parameters of PSI. The relationship between the effective quantum yield of PSI [Y(I)] and the non-photochemical quantum yield due to the donor side limitation of PSI [Y(ND)] (A-C), and between Y(I) and the non-photochemical quantum yield due to the acceptor side limitation of PSI [Y(NA)] (D-F) under low (LN, A, D), moderate (MN, B, E) and high nitrogen levels (HN C, F). For other details, see the legend of Fig. 2.

**Supplementary Figure S6**

Relationships between electron transport rate (ETR) and leaf N content per leaf area in mature leaves of rice plants. The data of ETRII (A) and ETRI (B) under the highest actinic light intensity were used. Pearson’s correlation coefficient and probabilities of statistical analysis are shown. For other details, see the legends of Fig. 2.
